# Pink beam crystallography demonstrated in SFX

**DOI:** 10.1107/S2052252521010794

**Published:** 2021-11-01

**Authors:** Takanori Nakane

**Affiliations:** a MRC Laboratory of Molecular Biology, Francis Crick Avenue, Cambridge Biomedical Campus, Cambridge, CB2 0QH, United Kingdom

**Keywords:** pink beams, serial femtosecond crystallography, XFELs, wide bandwidths

## Abstract

Nass *et al.* [ *IUCrJ* (2021), **8**, 905–920] applied a wide bandwidth beam (pink beam) to serial femtosecond crystallography at X-ray free electron lasers. This approach will lead to better datasets in a shorter time from fewer crystals.

Serial femtosecond crystallography (SFX) utilizes short and intense pulses from X-ray free-electron lasers (XFELs). The very short nature of the XFEL pulses, only a few tens of femtoseconds, enables tracking of fast reactions in time-resolved studies. It also allows the collection of practically damage-free structures, because atoms cannot move much in such a short period. This technique has been providing deep insights into reaction mechanisms of chemically and biologically interesting proteins such as photosystems and bacteriorhodopsin (Brändén & Neutze, 2021 ▸).

Unfortunately, as well as offering advantages, the short and bright XFEL pulses have disadvantages. Reconstruction of a three-dimensional map requires views from multiple directions. However, diffraction patterns in SFX are still images, because crystals do not have time to move during short exposures. In addition, intense XFEL pulses destroy crystals in one shot, precluding the collection of multiple images from a single crystal. Therefore, one has to collect diffraction patterns from thousands of crystals in different orientations and merge them to complete a dataset. This is in stark contrast to the rotation method, the typical data collection mode at home sources and synchrotron radiation facilities. In the rotation method, a crystal is rotated during exposure and multiple images are collected from a crystal. Accordingly one can obtain more information per crystal with this method. The article by Nass *et al.* in this issue of **IUCrJ** (Nass *et al.*, 2021 ▸) makes a significant contribution to addressing this problem.

The diffraction geometry is described by the so-called ‘Ewald construction’ in the reciprocal space [Fig. 1 ▸(*a*), also see the *Online Dictionary of Crystallography* (IUCr, 2021 ▸) for details]. The radius of the Ewald sphere is the inverse of the X-ray wavelength. As a crystal rotates, its associated reciprocal lattice rotates with the crystal. When a reciprocal lattice point intersects with the surface of the Ewald sphere, the point satisfies the Bragg condition and diffraction occurs.

In the rotation method, most reflections pass through the surface of the Ewald sphere over several images [Fig. 1 ▸(*b*)]. A data processing program integrates contributions from successive images to obtain full reflection intensities. In contrast, this is impossible in SFX, where a crystal does not rotate at all. The fraction of diffraction intensities recorded on an image is called partiality [Fig. 1 ▸(*c*)]. Multiple observations of a Bragg spot have different partialities depending on crystal orientation: some reflections only graze the Ewald sphere and have low partialities, while others fully intersect the surface and have high partialities. Advanced post-refinement algorithms can theoretically estimate partialities and scale partial intensities to the full intensity for merging (Ginn *et al.*, 2015 ▸; Sauter, 2015 ▸) but this is numerically unstable and does not always work in practice. A high multiplicity is often necessary to average out different partialities and obtain accurate structure factors. From an experimenter’s point of view, SFX requires higher numbers of crystals and longer data collection times than the rotation method.

Is there a way to obtain more information from a crystal without rotating it? A wide-bandwidth beam (also called a pink beam) provides one solution. With a pink beam, diffraction occurs when a reciprocal lattice point satisfies the Bragg condition for any wavelengths within the spectrum. We therefore consider intersection with a thick shell, instead of a thin surface, of the Ewald sphere(s); the thickness reflects the bandwidth of the polychromatic beam [Fig. 1 ▸(*a*)]. More reflections satisfy the Bragg condition. Moreover, partialities are improved, because reflections intersect with a thicker shell [Fig. 1 ▸(*d*)].

In Nass *et al.* (2021 ▸), the authors compared datasets collected at SwissFEL with different bandwidths (0.17 and 2.2%). The authors compared the dataset quality by performing experimental phasing. Experimental phasing requires the detection of tiny differences between Bijvoet pairs and is thus very sensitive to random and systematic errors in data acquisition and processing. Gratifyingly, in comparison with the 0.17% bandwidth dataset, the dataset with 2.2% bandwidth led to successful structure determination from about half the number of indexed diffraction patterns (50 000 versus 102 000). This confirms that the wider bandwidth enabled better convergence in data merging. In practical terms, this means that one can obtain more and/or higher resolution datasets within a limited beam time. This will facilitate time-resolved studies of difficult targets, where one needs to analyze many time points and/or detect low occupancy excited states.

Next, the authors re-processed the wide-bandwidth dataset with *pinkIndexer*, which offers new indexing and prediction refinement algorithms specifically designed for wide-bandwidth crystallography (Gevorkov *et al.*, 2020 ▸). This led to a higher indexing rate and further improved data quality, as shown by the fact that only 30 000 indexed images were sufficient for structure solution.

Interestingly, the data quality at high-resolution shells was worse in the wide-bandwidth datasets when comparison was made at the minimum number of the indexed patterns necessary for phasing. The authors attributed this paradoxical result to the fact that fewer photons contributed to a Bragg spot at high resolution. Future studies should verify this hypothesis by using a higher dose; fortunately, this should not be difficult with the high dynamic ranges of modern XFEL detectors.

While demonstrating the benefit of pink beams, this paper shows there is plenty of scope for further developing and refining the data processing algorithms. For example, the shape of diffraction spots differs from reflection to reflection but the current version of *CrystFEL* uses a circular integration mask of a fixed size. This is not suitable for radially elongated spots in pink beam crystallography. With better modeling of spot profiles, one can capture diffracted intensities better, while avoiding noise in neighboring pixels. Besides, most XFEL beamlines are equipped with an inline spectrometer that records shot-to-shot variations of XFEL spectra. This information should be stored as metadata together with the images and utilized in data processing, most importantly by post-refinement and partiality correction algorithms.

Finally, the authors should be applauded for depositing the raw diffraction images in CXIDB, a public database. This will enable method developers to use the data to improve data processing software. Users of the technique can also use the deposited images to practice data processing before their beam time.

## Figures and Tables

**Figure 1:**
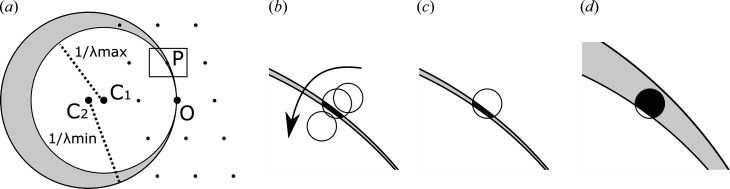
The Ewald sphere construction for a beam with a bandwidth. (*a*) O is the origin of the reciprocal space. C_1_ and C_2_ are two Ewald sphere centers corresponding to the longest (λmax) and the shortest (λmin) wavelengths in the spectrum, respectively. When a reciprocal lattice point (shown as small dots) is located within a shell bounded by the two Ewald spheres (shaded gray), the Bragg condition is satisfied and diffraction occurs. P is one of the diffracting reciprocal lattice points. The region near P is magnified in (*b*), (*c*) and (*d*). (*b*) With a narrow bandwidth beam, the two Ewald spheres are very close and the shell is thin. In the rotation method, the reciprocal lattice point sweeps through the surface of the Ewald sphere as the reciprocal space rotates with the crystal. One can measure the full intensity. (*c*) In a still shot, only a fraction of the reciprocal lattice point (shaded black) can be excited; thus, the partiality is low. (*d*) With a wide-bandwidth beam, the shell is thicker. Thus, the partiality is higher even with a stationary crystal.
